# Enhancing Human Cognitive Performance Through Physical Activity: Effect of Moderate-to-Vigorous Physical Activity on Executive Function in Chinese Adolescents

**DOI:** 10.3390/jintelligence14080186

**Published:** 2026-08-12

**Authors:** Yuan Liu, Chenyang Zhao, Zheping Gu, Hong Jun, Yanyan Hu, He Liu, Xiaojian Yin

**Affiliations:** 1 Physical Education College, Shanghai University, Shanghai 200444, China; cyzhao1019@163.com (C.Z.); guzp1220@163.com (Z.G.); 2 Key Laboratory of Adolescent Health Assessment and Exercise Intervention of the Ministry of Education, East China Normal University, Shanghai 200241, China; hongj9906@163.com (H.J.); yyhu1988@126.com (Y.H.); spaceliuhe@163.com (H.L.); xjyin1965@163.com (X.Y.); 3 College of Physical Education and Health, East China Normal University, Shanghai 200241, China; 4 Department of Physical Education, Shanghai Institute of Technology, Shanghai 201418, China

**Keywords:** exercise intervention, moderate-to-vigorous physical activity, human cognitive performance, executive function, adolescents

## Abstract

Executive function (EF) is a core component of human cognitive performance. This study investigated whether increasing the duration of moderate-to-vigorous physical activity (MVPA) could enhance this critical cognitive ability in Chinese adolescents. Adolescents (13.4 ± 0.5 years) from two secondary schools in Jiangsu Province were recruited, screened, and individually randomized into an intervention group (*n* = 101) and a control group (*n* = 105); after three withdrawals, the final analytical sample comprised 98 and 105 participants, respectively. EF was compared using independent and paired sample *t*-tests, analysis of covariance (ANCOVA), and effect size tests. Post-intervention, adolescents in the intervention group showed a significantly longer daily MVPA duration than the control group (mean difference 25.16 min, *p* < 0.05). As an exploratory analysis, sex-stratified ANCOVAs were conducted. After adjusting for pre-intervention inhibition control reaction times (RTs), ANCOVA revealed that boys in the intervention group had a lower mean inhibition control RT (1.03 ms lower than controls, *p* < 0.05). With pre-intervention 2-back RTs as a covariate, ANCOVA indicated significant reductions in mean 2-back RTs for intervention group boys (191.09 ms lower, *p* < 0.05) and girls (175.62 ms lower, *p* < 0.05). After adjusting for pre-intervention cognitive flexibility RTs, boys in the intervention group also exhibited a significantly lower mean cognitive flexibility RT (102.7 ms lower, *p* < 0.05). In conclusion, a combined aerobic and resistance (HIIT) exercise intervention effectively increased adolescents’ physical activity time, and concurrent improvements were observed in key components of human cognitive performance—specifically inhibition control, working memory, and cognitive flexibility in boys and working memory in girls.

## 1. Introduction

Inhibition control, working memory, and cognitive flexibility represent the three core subdomains of EF, a foundational element of human cognitive performance. These processes directly support cognitive performance by enabling individuals to maintain task-relevant focus, update and manipulate information, and flexibly shift between strategies—skills that are essential for academic learning and daily problem-solving (Meruelo et al., 2024). EF plays a crucial role in adolescents’ daily performance, learning abilities, and interpersonal interactions (Miyake et al., 2000). Numerous studies have indicated that adolescence is a critical period for the development of EF, which is influenced by various factors. Physical activity is an important and modifiable factor affecting EF (Diamond, 2013). Research has shown a close relationship between physical activity and EF in adolescents, suggesting that increasing physical activity levels may be an effective intervention to improve EF (Hillman et al., 2008). A daily minimum of 60 min of MVPA is recommended by the World Health Organization (WHO) for the 5- to 17-year-old population. However, 81% of children and adolescents fail to meet this guideline (Guthold et al., 2020). Beyond its direct benefits, physical activity may also serve as a protective factor against modern risk factors that impair EF. For instance, recent evidence suggests that physical activity moderates the negative association between short video addiction and EF in youth, potentially by counteracting the sleep-disrupting effects of excessive screen time (Xiao et al., 2026). In recent years, intervention studies about the effects of physical activity on EF have been increasingly conducted (Hillman et al., 2008). While most previous studies support the notion that physical activity can effectively enhance adolescents’ EF (Li et al., 2023), a minority of scholars disagree with this view (Pindus et al., 2016) and findings on the impact of physical activity on adolescents’ executive function remain inconsistent. These discrepancies may stem from factors such as participant characteristics, methods of assessing physical activity, choice of executive function paradigms, type of exercise intervention, and control of extraneous variables (Pindus et al., 2016). Previous research has found that different types of exercise produce varying effects on EF. However, most studies in this field have primarily focused on interventions involving single exercise modalities, with relatively few investigating combined exercise interventions (Li et al., 2023). Hillman et al. demonstrated that physical activity derived from aerobic exercise has beneficial effects on EF (Hillman et al., 2008). Barha et al. further compared three types of exercise interventions—aerobic training, resistance training, and multimodal training (aerobic + resistance training)—and found that aerobic training was more effective than resistance training in improving EF, while aerobic training alone was less effective than combined aerobic and resistance training. Based on these findings, this study adopts the combined exercise intervention model (Barha et al., 2017). Physical activity is the most important modifiable factor influencing EF. Further research has revealed that, compared to light physical activity (LPA), approximately 60 min of MVPA yields the optimal dose–response effect on EF (Liu, 2024). Therefore, this study employs a 16-week combined aerobic and resistance training intervention in adolescents to examine the effects of increased physical activity on EF and to explore effective strategies for promoting the healthy development of EF among adolescents. By testing whether a practical, school-based exercise intervention can improve EF, this study will provide empirical evidence on whether cognitive performance can be enhanced through behavioral interventions during adolescence.

Based on the literature reviewed above, this study aims to address the following research question: does a 16-week, school-based combined aerobic and resistance training intervention significantly improve the core components of EF—namely, inhibition control, working memory, and cognitive flexibility—in Chinese adolescents? We hypothesized that the intervention group would demonstrate significant pre-to-post improvements across all three EF components compared with the control group. Additionally, as an exploratory analysis, we examined sex-stratified patterns to identify potential differences worthy of further investigation without formulating a directional hypothesis, given the preliminary nature of existing evidence in this area.

## 2. Materials and Methods

### 2.1. Data Sources and Participant Recruitment

This study recruited first-grade boarding students from two junior high schools. Prior to enrollment, written informed consent was obtained from all participants and their parents. Eligibility was determined based on the following criteria: (1) intelligence quotient (IQ) ≥ 90 as measured by the Wechsler Intelligence Scale (Psychological Corporation, San Antonio, TX, USA); (2) absence of chronic medical conditions such as hypertension or color vision deficiency, as indicated by teacher reports; and (3) no involvement in high-level or professional athletics. After screening, eligible participants were assigned to the intervention group, which received a 16-week combined aerobic and resistance training program in addition to regular physical education, while the control group engaged in reading and writing activities. Additionally, to ensure data quality in the EF assessments, participants whose overall accuracy on any of the three EF tasks fell below 80% were excluded from the corresponding RT analyses. This threshold is widely used in reaction time research to confirm that participants adequately understood and complied with task instructions (Miller, 2023). Accuracy was therefore used primarily as a manipulation check; after excluding low-accuracy participants, reaction time served as the main dependent measure of EF performance. In the present study, all participants who completed the EF tasks met this criterion; therefore, no participants were excluded for this reason.

**Figure 1:**
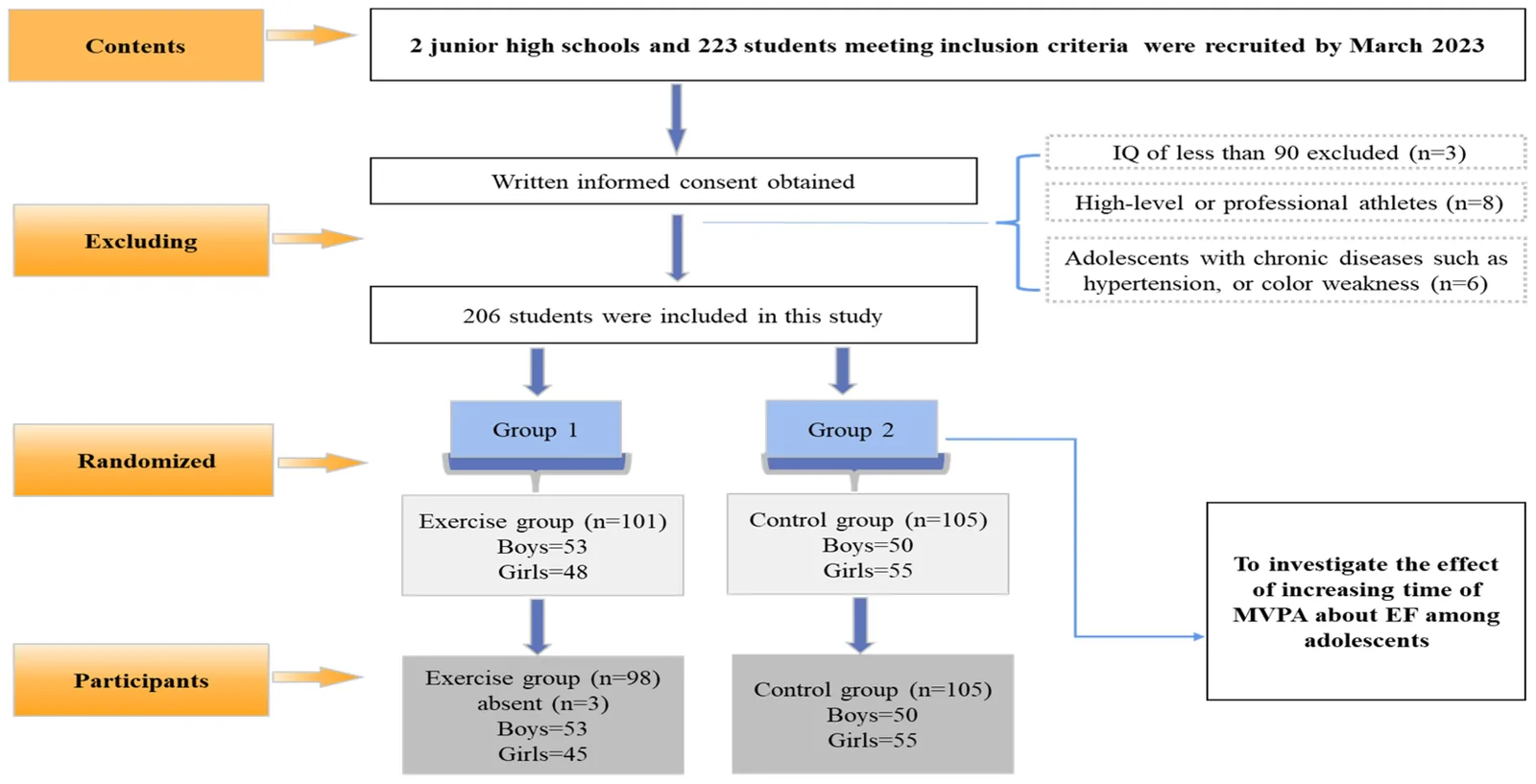
Flow diagram of participant selection and assignment. Abbreviations: IQ, intelligence quotient; MVPA, moderate-to-vigorous physical activity; EF, executive function.

Following the eligibility criteria described above, a total of 223 boarding students who provided written informed consent were assessed. Seventeen individuals (3 with IQ below 90, 8 high-level or professional athletes, and 6 with chronic medical conditions) were excluded, yielding 206 eligible participants who were then individually randomized using a computer-generated random number sequence into the intervention group (*n* = 101) and the control group (*n* = 105). The allocation sequence was generated by an independent statistician, concealed in sealed opaque envelopes, and not disclosed until baseline measurements were completed. During the 16-week intervention period, three participants in the intervention group withdrew due to prolonged school absence or school transfer; all control participants completed the study. Thus, the final analytical sample consisted of 98 adolescents in the intervention group and 105 in the control group. The gender and age distribution of the final sample is presented in Table 1. Outcome assessors responsible for cognitive testing and accelerometer data processing were blinded to group assignment throughout the trial (Figure 1).

Ethical clearance for this investigation was granted by the Human Research Ethics Committee of East China Normal University (approval No. HR761-2022), with all procedures strictly adhering to the ethical standards set forth in the Declaration of Helsinki. Prior to the commencement of survey administration, each participant received a comprehensive briefing regarding the study requirements. To guarantee privacy protection, all personal identifying information was converted into numerical codes rather than directly recorded.

### 2.2. Executive Function and Related Assessments

Inhibition control, working memory, and cognitive flexibility were assessed using three computerized tasks from the EF task-cuing paradigm (Chen et al., 2015): the Eriksen flanker task for inhibition control (Wylie et al., 2007), the N-back task for working memory (Smith & Jonides, 1997), and the more-odd shifting task for cognitive flexibility (Salthouse et al., 2003). Reaction time (RT) served as the primary outcome measure for all tasks, with shorter RTs indicating superior performance. Inhibition control was indexed by the RT difference between incongruent and congruent trials. Working memory was evaluated through 1-back and 2-back conditions. Cognitive flexibility was measured as the RT difference between heterogeneous and homogeneous trials. All tasks were implemented using E-prime 1.1 (Psychology Software Tools Inc., Pittsburgh, PA, USA).

For the Flanker task, participants completed 100 trials divided into four blocks, with 50 congruent and 50 incongruent trials presented in random order. For the N-back task, participants completed 1-back and 2-back conditions, each consisting of 40 trials, with stimuli presented in a fixed pseudo-random order. For the more-odd shifting task, participants completed 48 trials across two conditions (heterogeneous and homogeneous). The order of the three tasks was fixed across all participants (Flanker first, followed by N-back, and then more-odd shifting) to maintain consistency. Prior to formal testing, participants completed a familiarization session with 10 practice trials for each task to ensure they understood the instructions. Only participants who achieved > 80% accuracy during practice proceeded to formal testing. Reaction time data were cleaned by removing trials with RTs shorter than 200 ms or longer than 2000 ms, as well as trials with incorrect responses.

The 2-back condition was selected as the primary working memory index because it imposes greater demands on updating and monitoring processes compared with the 1-back condition, making it more sensitive to detecting intervention-related changes in working memory capacity (Smith & Jonides, 1997). The 1-back condition was used primarily as a practice/control task and is therefore not reported in the main results. Unlike inhibition control (calculated as the RT difference between incongruent and congruent trials) and cognitive flexibility (calculated as the RT difference between heterogeneous and homogeneous trials), working memory is presented as raw 2-back RT because the 1-back and 2-back conditions differ fundamentally in task difficulty, making a difference score less meaningful as a unitary index of working memory capacity. All cognitive assessments were administered by trained research assistants who were blinded to group allocation.

### 2.3. Physical Activity

#### 2.3.1. Objective Measurement Methods of Physical Activity

The ActiGraph GT3X+ three-axis accelerometer (ActiGraph, Pensacola, FL, USA) was used as the objective measurement instrument for physical activity levels. Prior to data collection, participants were instructed on the proper wearing procedures both online and in person. The accelerometer was worn at the waist and initialized to record at a 5 s sampling interval over a continuous period of at least 7 days. Participants were required to wear the device throughout the day and were asked to remove it only during bathing or swimming, as the device is not waterproof. After the 7-day wearing of the accelerometer was completed, the researchers collected the accelerometers uniformly and used the ActiLife version 6.10.2 (ActiGraph, Pensacola, FL, USA) software to download, process, and analyze the collected data. Among them, the valid data were tested, and the participants were required to have at least 4 days (3 school days + 1 weekend day) in their 7-day cycle, and the daily wearing time of the accelerometer was more than 600 min (Hillman et al., 2008). Using the cut-off points of Evenson et al. (Evenson et al., 2008), the time of SB, LPA, MPA, and VPA (min) was calculated. The results showed that the cut-off values of Evenson et al. had high validity in evaluating the physical activity of adolescents (Trost et al., 2011).

Non-wear time was defined as at least 60 consecutive minutes of zero counts, with an allowance of up to 2 min of non-zero counts. The vertical axis (counts per minute) was used for analysis, as it is the most commonly reported axis for youth physical activity studies. A valid day was defined as at least 600 min (10 h) of wear time. Participants were required to have at least 4 valid days (3 school days + 1 weekend day) out of the 7-day measurement period. The number of participants with valid accelerometer data was 203 (100%) at both pre-test and post-test. School days and weekend days were analyzed separately and then combined to calculate the average daily MVPA, as recommended for adolescent populations.

#### 2.3.2. Monitoring of the Intensity of Physical Activity Intervention

**Figure 2:**
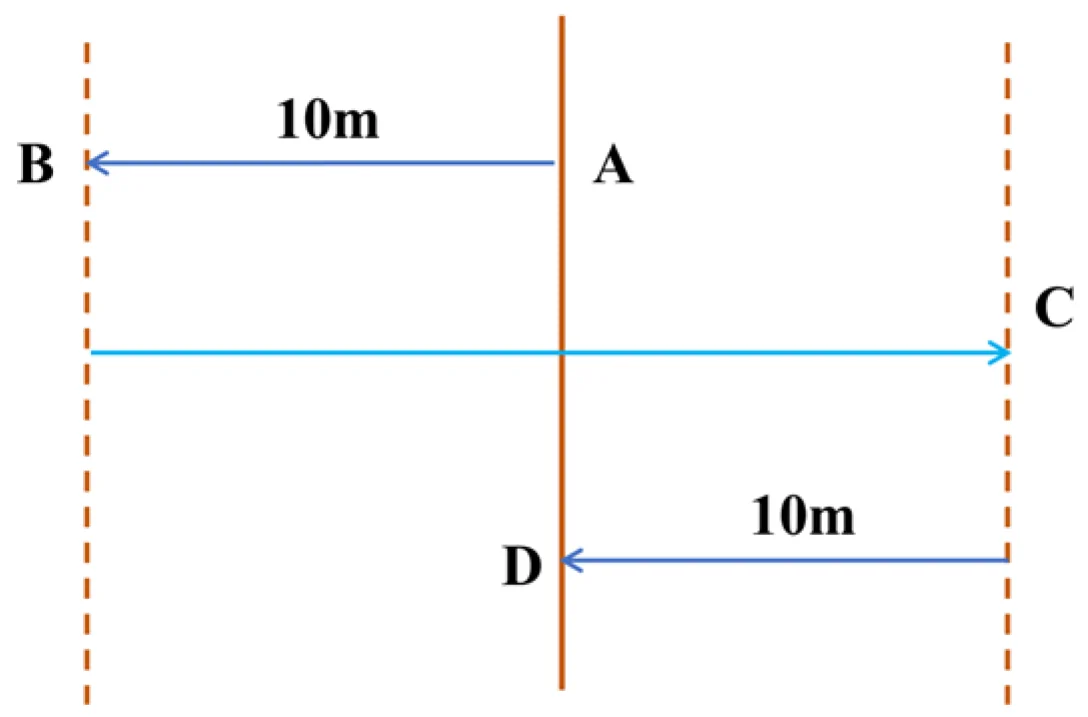
Schematic diagram of HIIT exercise for teenagers. (A) Starting point; (B) first turning point; (C) maximum sprint segment (20 m); (D) end point. Arrows indicate the running direction. One complete circuit from A → B → C → D constitutes one repetition.

In terms of the intensity control of aerobic exercise in High Intensity Interval Training (HIIT), according to the definition by Weston et al. (Weston et al., 2014), HIIT is characterized by an intensity of 85% to 95% of the maximal heart rate (HRmax), and this range was adopted in the present study. The intervention protocol was designed based on the research of Buchan et al. and adapted according to the characteristics of the participating adolescents, with input from sports and medical experts; it has been widely used in prior studies and has demonstrated good reliability and validity (Figure 2) (Buchan et al., 2012). The specific method: Teenagers in the HIIT group completed a 40 m fast run (including a 20 m maximum sprint, Part C) within 30 s. The starting point is at point A. The participants were required to start from point A, run to point B, then turn around and sprint at maximum speed towards point C, and then turn around and run again towards point D. The total distance was 40 m, which was one exercise session. The intensity was determined using the formula HRmax = 220 − age (years), and a Polar watch (Polar Electro Oy, Kempele, Finland) was used for real-time heart rate monitoring. Regarding the intensity control of resistance exercise, the percentage of each person’s maximum repetition (Repetition Maximum, RM) for each exercise was used to develop the resistance exercise load chart (Earle & Baechle, 2021).

Considering that the subjects were junior high school students and following the principle of gradually increasing the intensity of resistance exercise, and given that the intervention experiment intensity was of medium to high intensity, the resistance exercise intensity was determined to be 60% to 80% 1 RM (Thompson et al., 2013).

The detailed intervention protocol is described as follows. The intervention program comprised 48 sessions over 16 weeks (three sessions per week, 60 min per session). Each session consisted of five components: (1) A 5 min warm-up using basic aerobic steps (jumping jacks, lunge jumps, back kicks, and kick–step combinations); (2) 30 min of HIIT, in which participants performed repeated 40 m shuttle runs (including a 20 m maximal sprint) within 30 s per repetition, with a 30 s rest between repetitions. Intensity was maintained at 85–95% of age predicted maximal heart rate (HRmax = 220 − age), which corresponded to 175–196 beats per minute for the participants, and was monitored in real time using Polar heart rate monitors; (3) 10 min of fun games, such as obstacle running or group “figure-8” rope skipping, selected based on weather conditions; (4) 10 min of resistance training, including exercises such as planks, burpees, push-ups, and squats, performed at 60–80%of each participant’s estimated one-repetition maximum (1RM), with 3 sets of 10–15 repetitions per exercise and 1 min rest between sets; and (5) a 5 min cool-down period involving whole-body muscle and ligament stretching accompanied by relaxing music. The exercise intensity was progressively increased every 4 weeks by adjusting the training load or repetitions based on individual progress. Each session was supervised by trained research assistants, with a student-to-instructor ratio of approximately 10:1. Attendance was recorded at each session, with a mean attendance rate of 92.3%. No adverse events related to the intervention were reported throughout the study period.

The control group participants attended regular physical education classes and, during the intervention period, engaged in self-directed reading or homework activities in a quiet classroom setting. These sessions were matched in duration (60 min, three times per week) to the intervention sessions. Participants were asked whether they engaged in any additional extracurricular physical activities during the study period to minimize potential contamination, and no significant extracurricular exercise was reported.

### 2.4. Statistical Analysis

Normality was checked through methods such as histogram and Q-Q plot. For normally distributed variables, independent-sample *t*-tests were used to compare physical activity levels and EF sub-components between the intervention and control groups, while paired-sample *t*-tests were used to examine pre-to-post changes within each group. The ANCOVA method was performed to control for pre-intervention scores and to evaluate intervention effects on inhibition control RT, 2-back RT, and cognitive flexibility RT. Effect sizes were reported as partial *η*^2^ and were interpreted as follows: partial *η*^2^ = 0.01 as a small effect, partial *η*^2^ = 0.06 as a medium effect, and partial *η*^2^ = 0.14 as a large effect. Data were entered using Epi Data 3.0 statistical software (EpiData Association, Odense, Denmark), and image processing was conducted with GraphPad Prism 8.0 (GraphPad Software, San Diego, CA, USA) and Origin Pro9.1 (OriginLab Corp., Northampton, MA, USA) software. All statistical analysis was performed using SPSS 26.0 (IBM Corp., Armonk, NY, USA), with a two-sided test level of α = 0.05. All analyses were conducted on the per-protocol sample, as the three withdrawals occurred during the intervention period and no post-test data were available for these participants. Because the remaining participants completed both pre-test and post-test assessments with no missing data, an intention-to-treat analysis was not applicable. In addition, sex-stratified analyses were exploratory and were not adjusted for multiple comparisons; these findings should be interpreted with caution.

## 3. Results

### 3.1. Descriptive Characteristics for Various Variables of Control and Intervention Group

As shown in Table 2, the study found that before the intervention, there were no statistically significant differences in age, waist circumference, BMI, and PFI between the intervention group and the control group (*p* > 0.05). Baseline comparisons of EF and MVPA between the intervention and control groups also showed no significant differences for any outcome variable (all *p* > 0.05), confirming pre-intervention equivalence across groups. 

### 3.2. The Impact of Exercise Intervention on SB, LPA, and MVPA

**Figure 3:**
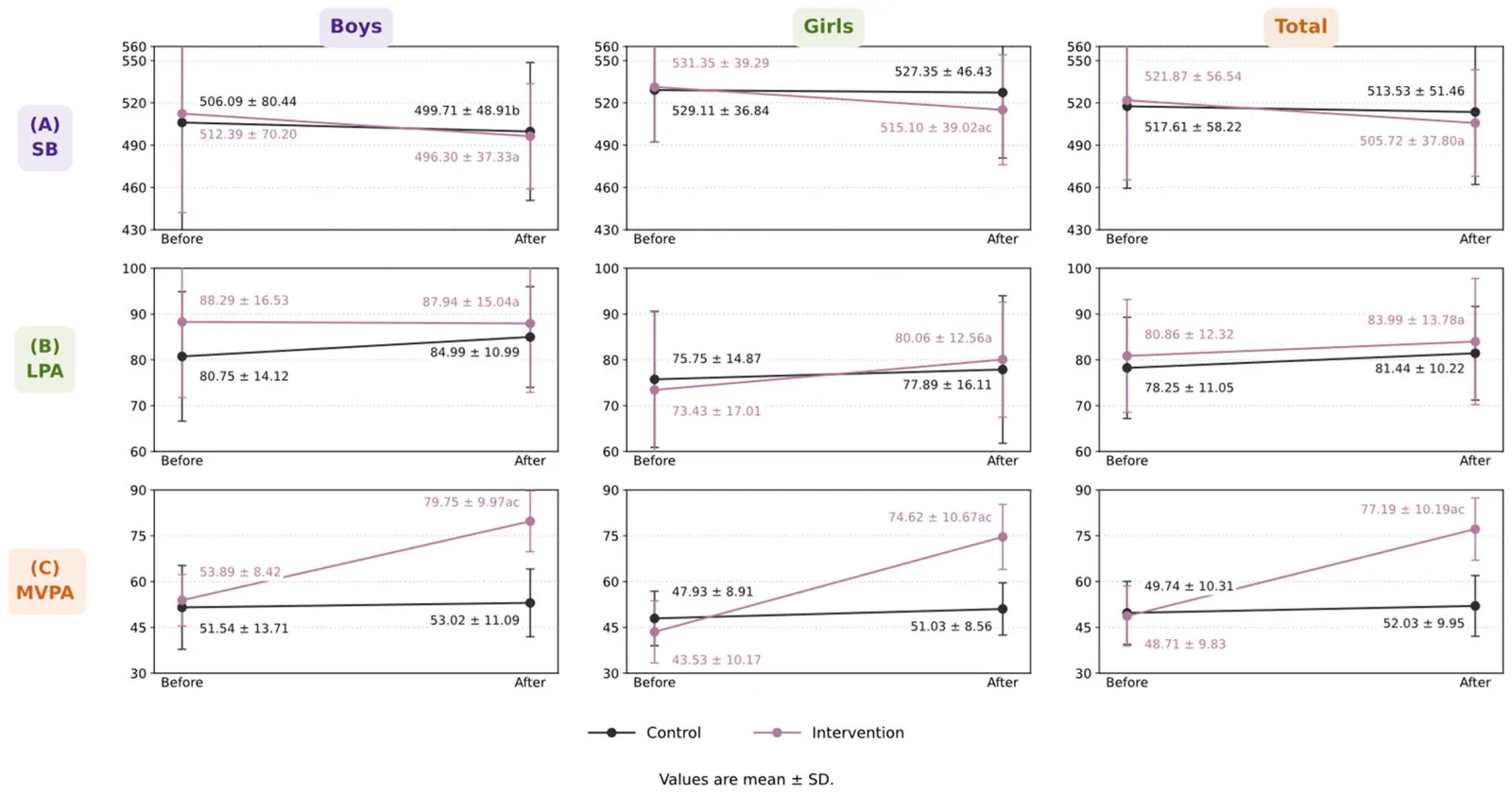
Trend charts of SB, LPA, and MVPA before and after intervention. Abbreviations: ^a^, comparison of the intervention group before and after the experiment is *p* < 0.05; ^b^, comparison of the control group before and after the experiment is *p* < 0.05; ^c^, comparison between the intervention group and the control group is *p* < 0.05.

As shown in Table 3 and Figure 3, the daily MVPA time of the adolescents in the intervention group after the intervention was 28.48 min higher than that before the intervention with a paired *t*-test (*p* < 0.05). Furthermore, independent-sample *t*-tests comparing the two groups post-intervention demonstrated that the intervention group exhibited a significantly higher daily MVPA duration than the control group, with a mean difference of 25.16 min (*p* < 0.05).

### 3.3. Impact of Exercise Intervention on RT in Executive Function Tasks

**Figure 4:**
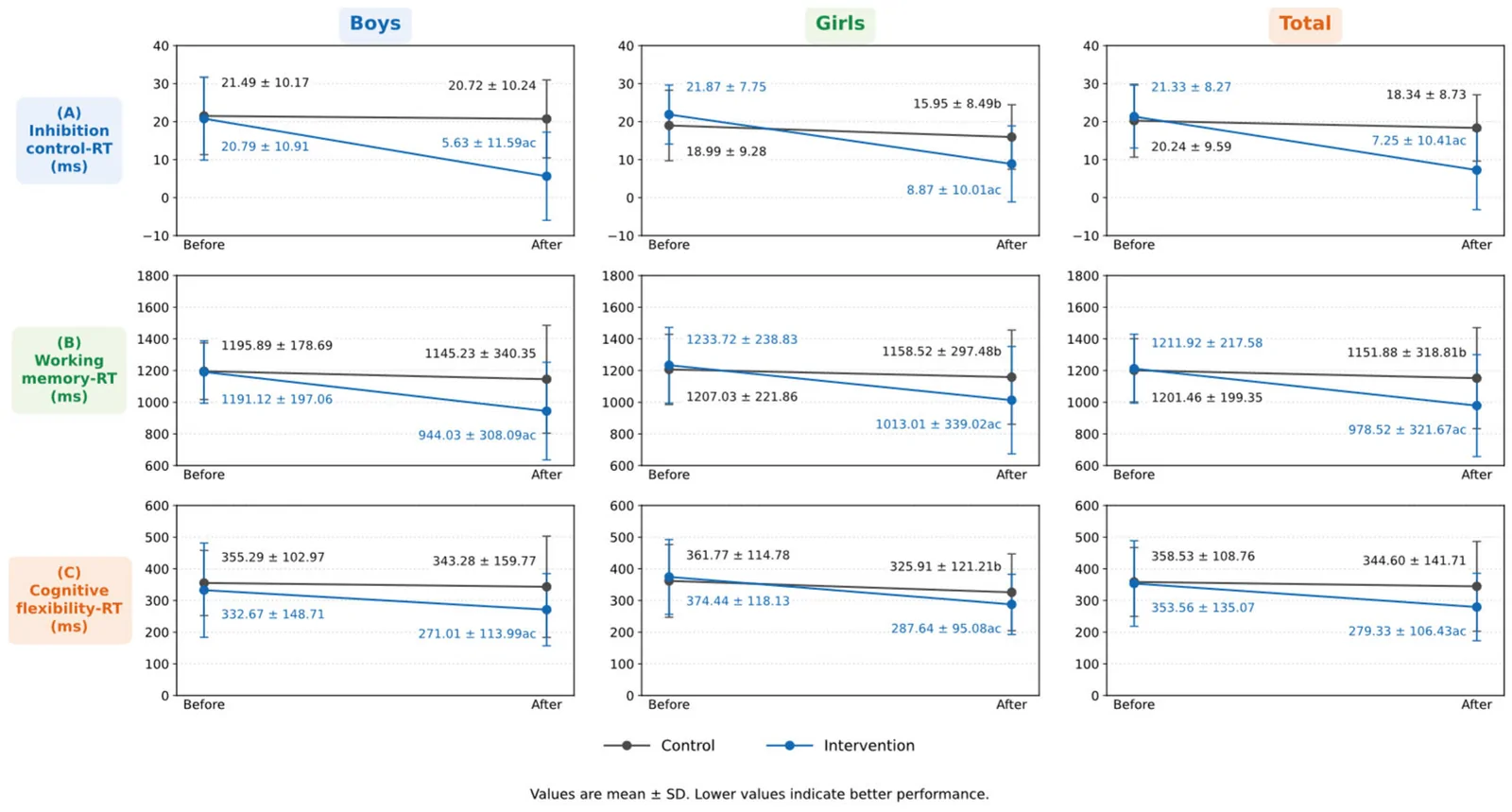
Trend chart of RT on executive function tasks before and after intervention. Abbreviations: ^a^, comparison of the intervention group before and after the experiment is *p* < 0.05; ^b^, comparison of the control group before and after the experiment is *p* < 0.05; ^c^, comparison between the intervention group and the control group is *p* < 0.05.

All participants who completed the EF assessments met the >80% accuracy criterion on each task; therefore, no participants were excluded from the RT analyses based on the accuracy threshold. Accuracy rates remained consistently high (>85%) across all tasks and time points, with no significant group differences (all *p* > 0.05), confirming that the observed RT reductions were not achieved at the expense of accuracy. As shown in Table 4 and Figure 4, using the independent sample *t*-test, the RT of inhibition control in the intervention group was 11.09 ms lower than that in the control group after the intervention (*p* < 0.05). A paired *t*-test further revealed that the intervention group’s inhibition control RT decreased by 14.08 ms from pre- to post-intervention (*p* < 0.05). Similarly, the intervention group showed a significantly lower 2-back-RT than the control group after the intervention, with a between-group difference of 173.36 ms (*p* < 0.05), and their post-intervention 2-back RT was 233.40 ms lower than their pre-intervention performance according to a paired *t*-test (*p* < 0.05). After the intervention, there was a significant difference in the cognitive flexibility RT between the control group and the intervention group (*p* < 0.05). A paired *t*-test further indicated that the intervention group’s cognitive flexibility RT decreased by 74.23 ms relative to their pre-intervention level (*p* < 0.05).

As shown in Table 5, ANCOVA on post-test corrected values of inhibitory control RT among boys, controlling for pre-test inhibition function RT, revealed that inhibitory control RT was lower in the intervention group than the control group among boys by 1.03 ms (F(1,100) = 6.485, partial *η*^2^ = 0.419, *p* = 0.031). For post-test corrected 2-back RT values, ANCOVA indicated a significant 191.09 ms reduction in the intervention group relative to the control group among boys (F (1,100) = 6.565, partial *η*^2^ = 0.04, *p* = 0.011), while girls in the intervention group exhibited a significant 175.62 ms lower 2-back RT than their control group counterparts (F (1,97) = 6.925, partial *η*^2^ = 0.037, *p* = 0.009). After adjusting for pre-test cognitive flexibility RT, ANCOVA yielded a significant overall group difference in cognitive flexibility RT between the intervention and control groups (*p* < 0.01). Stratified by gender, boys in the intervention group had a significantly lower cognitive flexibility RT than the control group by 102.7 ms (F (1,100) = 12.493, partial *η*^2^ = 0.153, *p* = 0.001). The sex-stratified analyses reported above are exploratory in nature and should be interpreted with caution.

## 4. Discussion

The exercise intervention program combining 60 min HIIT aerobic training and resistance training adopted in this study was associated with increases in PA levels, and concurrent improvements were observed in inhibition control, working memory, and cognitive flexibility in boys and working memory in girls (*p* < 0.05). When these results were further examined by sex, we observed a pattern in which boys appeared to benefit across all three EF components, whereas girls tended to show improvements primarily in working memory. These stratified observations are preliminary and warrant further investigation. There are relatively few studies on the combined exercise intervention program of aerobic training and resistance training for the physical activity and EF of adolescents, and there are certain differences in the previous research results. The reason for this might be that the research results of exercise intervention are inconsistent due to the influence of related factors such as different types of intervention, the selection of subjects, physical activity and the measurement methods of EF (Li et al., 2023). The following is a detailed analysis and discussion of the intervention effects on each sub-function of physical activity and EF in adolescents.

### 4.1. Intervention Analysis of Physical Activity and Inhibition Control

The positive effect of physical activity on inhibition control observed in this study aligns with findings from previous research (Ben-Zeev et al., 2020; Kao et al., 2023; G. X. Zhang, 2019; Q. J. Zhang, 2020). However, some studies have reported inconsistent findings (Leahy et al., 2020; Ludyga et al., 2019). The discrepancies in findings may be attributed to several methodological differences, including variations in physical activity assessment methods, cognitive paradigms (e.g., Stroop vs. Flanker tasks), and participant selection criteria. Additionally, the control group showed some pre-to-post reductions in RT (Table 4) which may reflect natural developmental maturation or practice effects from repeated task exposure. Nevertheless, the significantly greater improvements in the intervention group, after adjusting for pre-test scores, suggest that the exercise intervention was associated with benefits beyond these confounding factors. To strengthen the validity of future research, it is recommended to standardize measurement protocols, control for confounding variables, and conduct longitudinal follow-up studies. Such efforts would provide more robust scientific evidence to support interventions aimed at enhancing inhibition control in adolescents.

Specifically, we now note that a standard deviation larger than the mean is not uncommon for difference scores in RT research, as subtracting two RT measures (incongruent–congruent) tends to amplify variability (Miller, 2023). We also acknowledge that the substantial reduction in the congruency effect observed in both boys and girls is a notable change, suggesting improved inhibitory efficiency, while cautioning that these findings should be interpreted cautiously and warrant confirmation in future studies with larger samples and multiple testing occasions.

### 4.2. Intervention Analysis of Physical Activity and Working Memory

Based on the analysis of previous studies and the results of this study, exercise intervention is beneficial to improving the physical activity level of adolescents, and concurrent enhancements were observed in the performance level of their working memory levels (Booth et al., 2014; Moreau et al., 2017; Tottori et al., 2019; Valkenborghs et al., 2022). In the present study, the intervention group showed a 191.09 ms reduction in 2-back RT among boys and a 175.62 ms reduction among girls, with large effect sizes (partial *η*^2^ = 0.04 and 0.037, respectively). These effect sizes are comparable to or larger than those reported in previous HllT-based interventions, indicating that the combined aerobic and resistance protocol may be particularly effective for enhancing working memory.

There are also previous research results that are contrary to those of this paper (Cooper et al., 2016; Leahy et al., 2020; Wassenaar et al., 2021). These inconsistencies may be attributed to differences in task paradigms (e.g., Corsi block vs. 2-back), intervention characteristics, and sample demographics. Future studies should systematically examine how these moderating variables influence intervention outcomes.

### 4.3. Intervention Analysis of Physical Activity and Cognitive Flexibility

This study was found that this exercise intervention program was associated with increased physical activity levels in adolescents, and concurrent improvements were observed in boys’ cognitive flexibility performance. The results of this study are basically consistent with most previous studies (Hillman et al., 2014; Wang, 2021). However, the effect was significant only among boys (102.7 ms reduction, partial *η*^2^ = 0.153), whereas girls did not show a statistically significant improvement. Notably, several large-scale studies have reported null effects of exercise interventions on cognitive flexibility in adolescents (Syväoja et al., 2014; Wassenaar et al., 2021; Moreau et al., 2017). One possible interpretation of this pattern is that it may partly reflect differences in baseline fitness, exercise engagement, or neurobiological sensitivity to HIIT, although this remains to be tested directly. The relatively modest effect size for cognitive flexibility compared with inhibition control and working memory also suggests that this EF component may be less malleable through short-term exercise interventions.

From a theoretical perspective, the present findings contribute to the growing literature on cognitive intelligence plasticity by showing that a key component of cognitive performance—EF—can be improved through a practical, non-pharmacological behavioral intervention. Previous work on cognitive training has often shown limited far-transfer effects (Melby-Lervag & Hulme, 2013). In contrast, our findings suggest that physical activity may offer a more ecologically valid approach to improving EF in adolescents, thereby supporting the view that cognitive performance is responsive to behavioral interventions during critical developmental periods.

### 4.4. Potential Explanations for Sex Differences

When examined separately by sex, boys in this study showed improvements across all three EF components following the intervention, whereas girls showed improvements primarily in working memory. Whether this pattern reflects a genuine sex difference or is attributable to other factors cannot be determined from the present data. With this uncertainty in mind, we offer the following considerations as tentative contextualization rather than definitive explanation.

First, sex-specific brain maturation during adolescence may play a role; the prefrontal cortex, which supports EF, develops along different timelines in males and females (Luna et al., 2004). Second, pubertal hormonal changes—particularly testosterone and estradiol—have been shown to influence synaptic plasticity and dopaminergic systems that are relevant to cognitive control (Herting & Nagel, 2012). Third, although the intervention protocol was identical for both sexes, boys may have engaged more vigorously in the HIIT and resistance exercises due to greater baseline interest or physical fitness, potentially leading to higher exercise intensity or adherence, which could contribute to the observed sex differences. However, given the exploratory nature of these subgroup analyses and the relatively small sample size within each sex, these findings should be interpreted with caution. Future studies with larger, sex-balanced samples are needed to further clarify the mechanisms underlying sex differences in exercise-induced EF improvements.

### 4.5. Novel Contributions, Practical Implications, and Future Directions

This study makes several novel contributions to the existing literature. First, while most previous intervention studies have focused on single exercise modalities, we adopted a HIIT protocol and demonstrated its effectiveness in improving multiple EF subdomains in Chinese adolescents. Second, by objectively measuring physical activity using accelerometers rather than self-report questionnaires, we provide more reliable evidence linking increased MVPA to enhanced EF. Third, our findings point to potential sex-specific patterns of improvement that merit further investigation.

From a practical standpoint, our results have direct implications for school-based physical activity programs. The 16-week combined exercise protocol used in this study is feasible within a school setting and could be integrated into physical education curricula to promote both physical fitness and cognitive development in adolescents. Additionally, the observed sex differences suggest that schools may consider designing tailored exercise programs for boys and girls to optimize cognitive benefits.

Placing our findings within a broader cognitive health framework, a recent study by Xiao et al. demonstrated that physical activity moderates the relationship between short video addiction and EF in youth, with sleep quality serving as a mediating pathway (Xiao et al., 2026). While their work focused on physical activity as a protective buffer against a risk factor, our study provides complementary evidence that directly increasing MVPA through a structured exercise intervention can enhance EF independently. Together, these findings suggest that physical activity may support adolescent cognitive health through multiple pathways—both by directly improving EF and by mitigating the negative impact of modern behavioral risk factors such as excessive screen time and sleep disruption.

Several directions for future research should be considered. Longitudinal follow-up studies are needed to determine whether the observed EF improvements are sustained over time. The present study focused on behavioral outcomes, so future research incorporating neuroimaging or biomarker assessments would help clarify the neural mechanisms underlying exercise-induced EF improvements. Finally, extending this intervention to high school students and other populations would help establish the generalizability of our findings across different developmental stages.

Theoretically, these findings support the view that cognitive performance is responsive to through behavioral interventions. By demonstrating that a 16-week combined exercise program was associated with improvements in EF in adolescents, this study provides empirical evidence that cognitive performance can be enhanced during a critical developmental window. This extends previous work on cognitive training, which has often shown limited transfer effects (Melby-Lervag & Hulme, 2013), by showing that physical activity may offer a more ecologically viable approach. Overall, our results contribute to the growing literature on cognitive enhancement by highlighting the potential of school-based physical activity interventions to promote cognitive performance in adolescents.

## 5. Strengths and Limitations

### 5.1. Strengths

On the one hand, prior research on the effects of physical activity interventions on EF in adolescents remains limited. Furthermore, this study integrates a high-intensity interval training protocol incorporating both aerobic and resistance exercise components to examine its impact on the subcomponents of EF (inhibition control, working memory, and cognitive flexibility).

### 5.2. Limitations

Several limitations should be acknowledged. First, although participants were individually randomized, they were recruited from only two schools, which may introduce some degree of nesting. While the individual-level randomization reduces clustering effects, future studies with larger samples drawn from more schools should consider multilevel or mixed-effects models to fully account for any residual clustering at the school or class level. The sex-stratified analyses were exploratory in nature and not supported by formal Group × Time × Sex interaction tests; future studies with larger, sex-balanced samples specifically designed to test sex as a moderating variable are needed to verify the observed sex-specific patterns. The absence of long-term follow-up also limits conclusions about the durability of the observed improvements, and dose–response relationships between exercise attendance and cognitive outcomes were not formally analyzed. Due to some inevitable factors, this study failed to provide aerobic and resistance combined exercise intervention for physical activity and EF to high school students. Whether the intervention conclusion for junior high school students has the same ecological effect in the senior high school stage remains to be further explored through intervention experiments on high school students in the future.

Furthermore, the present study focused on behavioral measures of EF and did not include neurobiological or physiological assessments (e.g., BDNF levels, cerebral blood flow, or prefrontal cortex activation via EEG/fMRI). While the observed improvements in EF are consistent with exercise-induced cognitive benefits reported in the literature, we are unable to directly test the underlying neural mechanisms. Future studies incorporating neuroimaging or biomarker assessments are warranted to clarify how exercise interventions affect brain function in adolescent populations. Additionally, no correction for multiple comparisons was applied to the exploratory sex-stratified analyses, and these findings should be interpreted with caution. Finally, the control group engaged in sedentary reading or homework activities during the intervention sessions, which served as a time-matched comparison but did not constitute an active control condition for attention, social interaction, or general arousal. Consequently, we cannot rule out the possibility that some of the observed improvements in the intervention group may be partially attributed to non-specific factors such as increased social engagement, expectation effects, or enhanced motivation, rather than the exercise itself. Future studies incorporating an active control condition (e.g., non-exercise group activities with similar social interaction and instructor attention) would help isolate the specific cognitive benefits attributable to physical activity.

## 6. Conclusions

The research found that the combined aerobic and resistance (HIIT) exercise intervention significantly increased the daily MVPA time of junior high school students; concurrent improvements were observed in inhibition control, working memory, and cognitive flexibility in boys and working memory in girls. These findings suggest that increasing MVPA through such a combined exercise intervention may serve as an effective strategy to enhance human cognitive performance is associated with EF in adolescents.

## Figures and Tables

**Table 1:** Gender and age of adolescents in the intervention and the control group.

Group	Gender	N (%)	Age		
			M ± SD	Minimum	Peak
Intervention	Boys	53 (54.08)	13.6 ± 0.4	12	15
	Girls	45 (45.92)	13.2 ± 0.5	13	14
	Total	98 (100)	13.4 ± 0.4	12	14
Control	Boys	50 (47.62)	13.6 ± 0.5	13	15
	Girls	55 (52.38)	13.5 ± 0.4	13	14
	Total	105 (100)	13.5 ± 0.5	13	15
In all		203 (100)	13.4 ± 0.5	12	15

**Table 2:** WC, BMI and PFI of the intervention and the control group before the test.

Gender	Group	WC	BMI	PFI
		(cm)	(kg/m^2^)	
Boys	Intervention group	74.25 ± 11.20	20.16 ± 3.86	2.03 ± 3.98
	Control group	71.64 ± 8.87	20.54 ± 3.66	1.51 ± 3.65
	Difference	2.61	−0.38	0.52
	Difference 95% CI	−1.471~6.687	−1.902~1.148	−1.033~2.061
	*T*	1.269	−0.491	0.66
	*P*	0.207	0.625	0.511
	Cohen’s *d*	0.259	0.100	0.135
Girls	Intervention group	70.28 ± 9.37	19.20 ± 3.76	1.29 ± 4.13
	Control group	66.23 ± 10.91	19.38 ± 3.72	1.25 ± 3.33
	Difference	4.05	−0.17	0.04
	Difference 95% CI	−0.094~8.184	−1.690~1.344	−1.475~1.555
	*T*	1.941	−0.226	0.053
	*P*	0.055	0.822	0.958
	Cohen’s *d*	0.396	0.046	0.011

Abbreviations: WC, waist circumference; BMI, body mass index; PFI, physical fitness index; CI, confidence interval.

**Table 3:** Comparison of SB, LPA, and MVPA between the control and the intervention group.

Subgroups			Boys	Girls	Total
			M ± SD	M ± SD	M ± SD
SB	Before	Control	506.09 ± 80.44	529.11 ± 36.84	517.61 ± 58.22
		Intervention	512.39 ± 70.20	531.35 ± 39.29	521.87 ± 56.54
	After	Control	499.71 ± 48.91 ^b^	527.35 ± 46.43	513.53 ± 51.46
		Intervention	496.30 ± 37.33 ^a^	515.10 ± 39.02 ^ac^	505.72 ± 37.80 ^a^
LPA	Before	Control	80.75 ± 14.12	75.75 ± 14.87	78.25 ± 11.05
		Intervention	88.29 ± 16.53	73.43 ± 17.01	80.86 ± 12.32
	After	Control	84.99 ± 10.99	77.89 ± 16.11	81.44 ± 10.22
		Intervention	87.94 ± 15.04 ^a^	80.06 ± 12.56 ^a^	83.99 ± 13.78 ^a^
MVPA	Before	Control	51.54 ± 13.71	47.93 ± 8.91	49.74 ± 10.31
		Intervention	53.89 ± 8.42	43.53 ± 10.17	48.71 ± 9.83
	After	Control	53.02 ± 11.09	51.03 ± 8.56	52.03 ± 9.95
		Intervention	79.75 ± 9.97 ^ac^	74.62 ± 10.67 ^ac^	77.19 ± 10.19 ^ac^

Abbreviations: ^a^, comparison of the intervention group before and after the experiment is *p* < 0.05; ^b^, comparison of the control group before and after the experiment is *p* < 0.05; ^c^, comparison between the intervention group and the control group is *p* < 0.05.

**Table 4:** Comparison of executive function between control and intervention group.

Subgroups			Boys	Girls	Total
			M ± SD	M ± SD	M ± SD
Inhibition control-RT	Before	Control	21.49 ± 10.17	18.99 ± 9.28	20.24 ± 9.59
		Intervention	20.79 ± 10.91	21.87 ± 7.75	21.33 ± 8.27
	After	Control	20.72 ± 10.24	15.95 ± 8.49 ^b^	18.34 ± 8.73
		Intervention	5.63 ± 11.59 ^ac^	8.87 ± 10.01 ^ac^	7.25 ± 10.41 ^ac^
Working memory-RT	Before	Control	1195.89 ± 178.69	1207.03 ± 221.86	1201.46 ± 199.35
		Intervention	1191.12 ± 197.06	1233.72 ± 238.83	1211.92 ± 217.58
	After	Control	1145.23 ± 340.35	1158.52 ± 297.48 ^b^	1151.88 ± 318.81 ^b^
		Intervention	944.03 ± 308.09 ^ac^	1013.01 ± 339.02 ^ac^	978.52 ± 321.67 ^ac^
Cognitive flexibility-RT	Before	Control	355.29 ± 102.97	361.77 ± 114.78	358.53 ± 108.76
		Intervention	332.67 ± 148.71	374.44 ± 118.13	353.56 ± 135.07
	After	Control	343.28 ± 159.77	325.91 ± 121.21 ^b^	344.60 ± 141.71
		Intervention	271.01 ± 113.99 ^ac^	287.64 ± 95.08 ^ac^	279.33 ± 106.43 ^ac^

Abbreviations: ^a^, comparison of the intervention group before and after the experiment is *p* < 0.05; ^b^, comparison of the control group before and after the experiment is *p* < 0.05; ^c^, comparison between the intervention group and the control group is *p* < 0.05.

**Table 5:** Covariance analysis of the post-test correction values of RT on executive function tasks after intervention.

Subgroups		Boys	Girls	Total
		M ± SE	M ± SE	M ± SE
Inhibition control-RT	Intervention	10.25 ± 7.54	10.68 ± 8.84	10.86 ± 8.26
	Control	11.28 ± 7.18	21.33 ± 11.37	18.16 ± 11.38
	*F*	6.485	0.135	0.552
	*p*	0.031 *	0.717	0.462
	Partial *η*^2^	0.419	0.005	0.014
Working memory-RT	Intervention	961.6 ± 422.10	975.94 ± 421.43	969.16 ± 420.47
	Control	1152.69 ± 315.10	1151.56 ± 318.93	1152.09 ± 316.26
	*F*	6.565	6.925	13.633
	*p*	0.011 *	0.009 **	0.000 **
	Partial *η*^2^	0.04	0.037	0.038
Cognitive flexibility-RT	Intervention	270.05 ± 114.15	312.57 ± 193.76	276.35 ± 127.49
	Control	372.75 ± 118.20	305.61 ± 199.77	348.77 ± 153.45
	*F*	12.493	1.608	6.972
	*p*	0.001 **	0.22	0.010 **
	Partial *η*^2^	0.153	0.078	0.07

Abbreviations: *, comparison of the modified measured values between the intervention group and the control group is *p* < 0.05, and **, comparison of the modified measured values between the intervention group and the control group is *p* < 0.01.

## Data Availability

The data presented in this study are available upon request from the corresponding author due to privacy or ethical restrictions (the study involved human participants, and the dataset contains potentially identifying information).
